# Preliminary evidence about the effects of meditation on interoceptive sensitivity and social cognition

**DOI:** 10.1186/1744-9081-9-47

**Published:** 2013-12-23

**Authors:** Margherita Melloni, Lucas Sedeño, Blas Couto, Martin Reynoso, Carlos Gelormini, Roberto Favaloro, Andrés Canales-Johnson, Mariano Sigman, Facundo Manes, Agustin Ibanez

**Affiliations:** 1 Laboratory of Experimental Psychology and Neuroscience (LPEN), INECO (Institute of Cognitive Neurology) and Institute of Neuroscience, Favaloro, Favaloro University, C1078AAI, Buenos Aires, Argentina; 2 UDP-INECO Foundation Core on Neuroscience (UIFCoN), Diego Portales University, Santiago, Chile; 3 Medical Research Council Cognition and Brain Sciences Unit, Cambridge, CB2 7EF, UK; 4 Physics Department, Laboratory of Integrative Neuroscience, FCEyN UBA and IFIBA, Conicet, Pabellón 1, Ciudad Universitaria, 1428 Buenos Aires, Argentina; 5 National Scientific and Technical Research Council (CONICET), Buenos Aires, Argentina; 6 Universidad Torcuato Di Tella, Almirante Juan Saenz Valiente 1010, Buenos Aires, C1428BIJ, Argentina; 7 Australian Research Council (ACR) Centre of Excellence in Cognition and its Disorders, New South Wales, Australia

**Keywords:** Interoception, Meditation, Mindfulness, Social cognition, Heartbeat detection task

## Abstract

**Background:**

Interoception refers to the conscious perception of body signals. Mindfulness is a meditation practice that encourages individuals to focus on their internal experiences such as bodily sensations, thoughts, and emotions. In this study, we selected a behavioral measure of interoceptive sensitivity (heartbeat detection task, HBD) to compare the effect of meditation practice on interoceptive sensitivity among long term practitioners (LTP), short term meditators (STM, subjects that completed a Mindfulness-Based Stress Reduction (MBSR) program) and controls (non-meditators). All participants were examined with a battery of different tasks including mood state, executive function and social cognition tests (emotion recognition, empathy and theory of mind).

**Findings:**

Compared to controls, both meditators’ groups showed lower levels of anxiety and depression, but no improvement in executive function or social cognition performance was observed (except for lower scores compared to controls only in the personal distress dimension of empathy). More importantly, meditators’ performance did not differ from that of nonmeditators regarding cardiac interoceptive sensitivity.

**Conclusion:**

Results suggest no influence of meditation practice in cardiac interoception and in most related social cognition measures. These negative results could be partially due to the fact that awareness of heartbeat sensations is not emphasized during mindfulness/vipassana meditation and may not be the best index of the awareness supported by the practice of meditation.

## Background

Interoception involves the conscious perception of feelings from inside the body [1-3]. Interoception has been proposed to modulate social cognition processes such as motivational behavior [2], empathy [4], and theory of mind (ToM), which have been suggested to be supported by emotional and body feedback information [4].

Meditation is a form of mental training [5] encouraging individuals to focus on their internal experiences, such as bodily sensations, thoughts, and emotions [6]. One component of meditation involves the development of interoceptive attention to visceral sensations [7]. Additionally, meditation practice promotes the development of prosocial behavior [8].

Previous findings reported no difference in interoception accuracy between meditators and nonmeditators [5,9]. In these studies, a heartbeat discrimination paradigm was selected: participants had to discriminate whether their heartbeats synchronized with either auditory or visual cues [10]. Consequently, subjects had to attend at the same time to their cardiac sensation and to external stimuli which have been shown to affect interoceptive performance [11]. We selected a different heartbeat detection paradigm [12] to avoid the possible interference of external stimuli. Moreover, given the relationship between interoception and social cognition [2,4,13,14], we included tasks of emotion recognition, empathy and ToM to test the association among bodily perception, social cognition and meditation practice. Moreover, considering the interaction between executive functions (EF) and social cognition domains (emotional processing [15], ToM [16] and empathy [17]), EF abilities were also evaluated.

Our aim was to compare the effect of meditation practice on interoceptive sensitivity and related measures among long term practitioners (LTP), subjects that completed a Mindfulness-Based Stress Reduction (MBSR) program (short term meditators, STM) and controls (nonmeditators). We predicted that long term practitioners would show enhanced interoceptive sensitivity, reflected in a better performance in heartbeat detection and related domains of social cognition.

## Methods

### Subjects

Ten nonmeditators, 9 short-term meditators and 10 long-term practitioners participated. The LTP group’s mean was 4.35 (SD = 2.17) years of continued practice and the STM completed an 8-week Mindfulness-Based Stress Reduction (MBSR) program (see criteria in the Additional file 1: Table S1). Controls had never attended a yoga or meditation course. Groups were age, gender and education matched. We controlled body mass index because it influences the interoceptive performance [18]. Participants had no history of drug abuse, neurological or psychiatric conditions. Participants provided an informed consent in accordance with the Declaration of Helsinki and the study was approved by the institutional ethics committee.

### Neuropsychological and clinical evaluation

Participants completed the Beck’s Depression Inventory (BDI) and the State Trait Anxiety Inventory (STAI) to evaluate mood and affective state, respectively. EF were assessed with the INECO Frontal Screening (IFS) [19] indexing 8 EF (see Additional file 1: Table S1) and the Stroop test.

### Social cognition tasks

A description of social cognition tasks (empathy, theory of mind and emotion recognition) is provided in Table 1 (see also Additional file 1: Table S1 for a detailed explanation of the materials and methods).

**Table 1:** Interoception and social cognition domain assessed and tasks employed

***Interoception***	***Task***	***Description***
**Interoception sensitivity**	HBD	The HBD is a motor tracking test that assesses interoception sensitivity. Participants had to tap a key on a keyboard along with their heartbeat in different conditions. First, as a motor-control condition, participants had to follow an audio-recording of a heartbeat. Next, they had to follow their heartbeat without external feedback (intero-pre condition). Then they had to do the same while receiving simultaneous auditory feedback of their own heart provided through online EKG register (feedback condition). Finally, they had to follow their own heartbeat without feedback (intero-post condition). These conditions offer a measure of audio-motoric performance (first condition), and a cardiac interoceptive measure (second and fourth conditions), prior to and after the feedback condition, respectively. During this task we also measured heart rate and heart rate variability to control their possible influence on interoception sensitivity; results showed no differences among groups (see Additional file 2).
***Social cognition domain***	***Task***	***Description***
**Emotional recognition**	Emotional morphing	This task assesses recognition of six basic emotion expressions and consists of 48 morphing faces randomly presented on a screen (see Additional file 1).
**Theory of mind**	ToM	This test assesses the emotional inference aspect of the ToM. Consist of 36 pictures of the eye region of a face. Participants chose which of four words best described the person’s thoughts or feelings in each picture.
**Empathy**	IRI	The IRI is a 28-item self-report questionnaire that separately measures both the cognitive and affective components of empathy.

*HBD*=Heartbeat Detection task; *ToM*=Theory of Mind; *IRI*=Index of Interpersonal Reactivity.

### Interoception

#### Heartbeat detection task (HBD)

The HBD is a motor tracking test that assesses interoception sensitivity [12]. Participants had to tap a key on a keyboard along with their heartbeat in different conditions (see Table 1 and the Additional file 1: Table S1 data for a more detailed explanation).

### Data analysis

Demographic, neuropsychological, and experimental data were compared among groups using ANOVA and Tukey’s HSD *post*-*hoc* tests. For categorical variables (e.g., gender), Kruskal-Wallis tests were applied. Mixed repeated measured ANOVA was performed for HBD, with a within-subject factor (the four conditions) and a between-subject factor (group).

## Results

### Demographic and neuropsychological results

No differences were found in gender [H = 4.90, p = 0.86], age [F (2, 25) = 0.95, p = 0.39, η_p_^2^ 0.07], formal education [F (2, 25) = 2.13, p = 0.13, η_p_^2^ = 0.14] or body mass index [F (2, 21) = 1.47, p = 0.25, η_p_^2^ =0.12] among groups.

Groups showed similar EF performance measured by the IFS [F (2, 25) = 1.50, p = 0.24, η_p_^2^ =0.10]. There were no differences in the three condition of the Stroop task, word [F (2, 23) = 0.20, p = 0.81, η_p_^2^ =0.01], color [F (2, 23) = 1.40, p = 0.26, η_p_^2^ =0.10] and incongruent color-word [F (2, 23) = 0.35, p = 0.70, η_p_^2^ =0.03]. No interference effect was found [F (2, 23) = 1.88, p = 0.17, η_p_^2^ =0.14] (See Table 2).

**Table 2:** **Demographic**, **neuropsychological and clinical results**

	**F**	**p**	**Controls**	**STM**	**LTM**
*Gender*	4.90 (H)	.86	2 M : 8 F	4 M : 4 F	7 M : 3 F
*Age* (*years*)	.95	.40	M= 37.30;	M= 41.12;	M= 43.80;
			SD= 9.12	SD= 12.15	SD=10.55
			(22 – 49)	(25 – 55)	(29 – 56)
*Formal education* (*years*)	2.13	.13	M=16.10;	M= 16.13;	M= 17.90;
			SD= .74	SD= 1.73	SD= 3.25
			(15 – 17)	(12 – 17)	(12 – 25)
*Body mass index*	1.47	.25	M= 22.94;	M= 22.88;	M= 24.87;
			SD= 2.75	SD= 3.15	SD= 2.49
			(19.43 – 26.67)	(17.63 – 26.51)	(21.60 – 28.57)
*IFS* (*Ineco Frontal Score*)	1.50	.24	M= 25.95;	M= 27.44;	M= 26.25;
			SD= 1.50	SD= 2.47	SD= 1.69
			(23 – 28)	(23 – 30)	(24 – 30)
*Stroop Interference score*	1.88	.17	M= 5.02;	M= 11.33;	M=5.36;
			SD= 8.06	SD= 7.20	SD= 7.03
			(-6.59 – 15.95)	(5.54 – 27.70)	(-2.64 – 15.85)
*BDI*-*II*	4.12	.03	M=9.90;	M=2.88;	M=3.50;
			SD=6.94	SD=2.30	SD=6.70
			(2 – 22)	(0 – 7)	(0 – 22)
*STAI Trait*	3.74	.03	M=40.30;	M=33.25;	M=30.30;
			SD=9.75	SD=6.54	SD=8.12
			(25 – 61)	(27 – 43)	(23 – 51)
*STAI State*	1.87	.17	M=34.20;	M=30.88;	M=27.90;
			SD=10.87	SD=2.70	SD=4.86
			(20 – 54)	(28 – 34)	(23 – 37)

*M*= mean.

*SD*= standard deviation.

Numbers within () are min-max.

### Clinical evaluation

We observed a significant difference for BDI score among groups [F (2, 25) = 4.12, p < 0.05,, η_p_^2^ = 0.24]. Post-hoc comparisons (Tukey HSD test, MS = 34.97; df = 25.00) revealed higher scores of depressive symptoms in controls compared to STM (p < 0.05). We did not observe between group differences for STAI-State subscale [F (2, 25) = 1.87, p = 0.17, η_p_^2^ = 0.13]. However, significant differences for STAI-Trait subscale [F (2, 25) = 3.74, p < 0.05, η_p_^2^ = 0.23] were observed; post hoc comparisons (Tukey test, HSD, MS = 69.98; df = 25.00) showed controls had significantly higher anxiety scores (p < 0.05) than LTM.

### Social cognition measures

**Figure 1:**
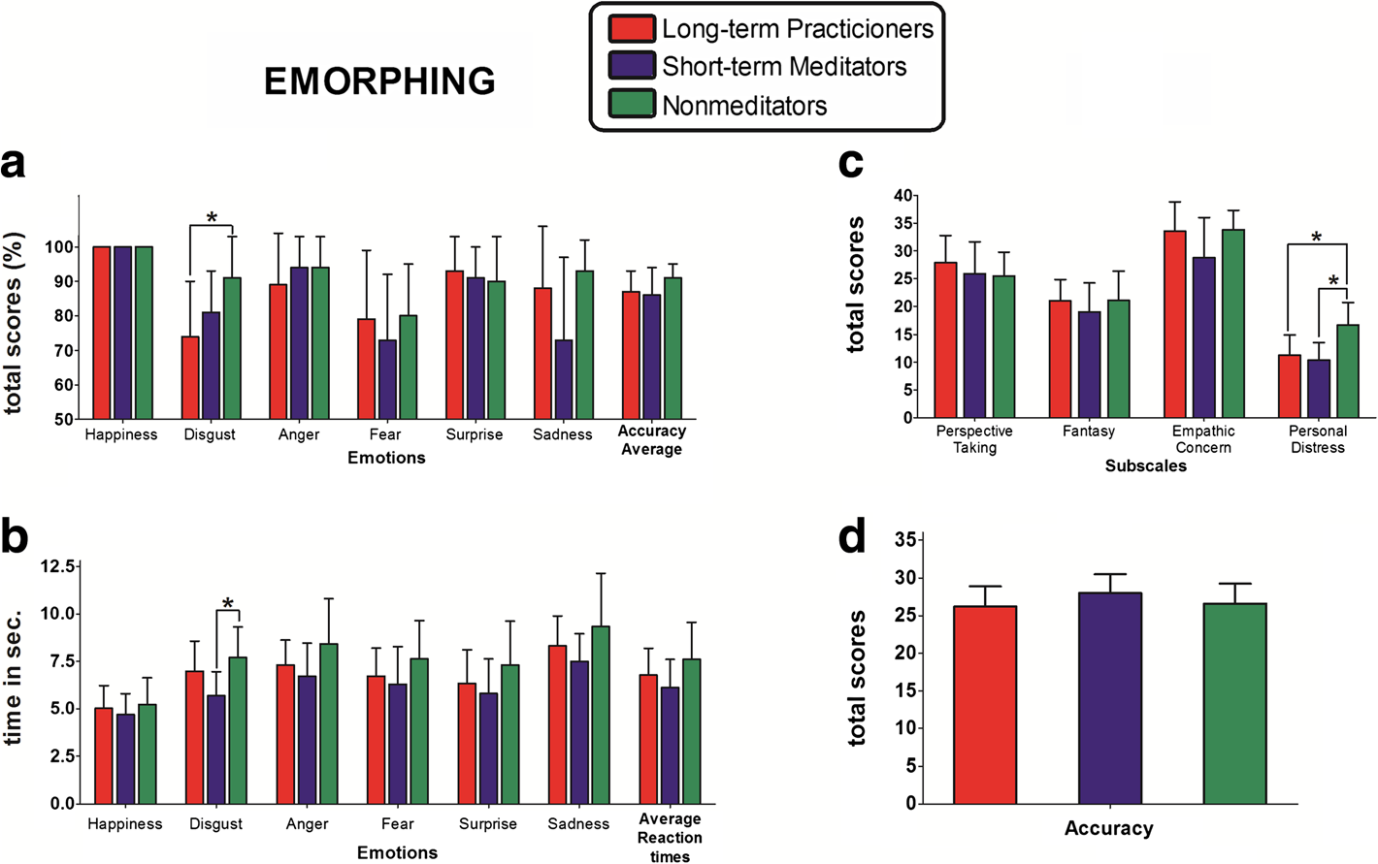
**Social cognition.** Emorphing. Percent of accuracy **(a)** and reaction times in seconds **(b)** are depicted for every basic emotion and for average scores. Interpersonal reactivity index (IRI). Raw scores of each subscales are presented **(c)**. Reading the mind in the eyes (ToM) Total scores **(d)**. *indicates significant differences.

Emotion recognition: No differences were observed regarding total accuracy [F (2, 25) = 2.49, p = 0.10, η_p_^2^ = 0.16]. However, per category analysis showed significant differences in disgust recognition among groups [F (2, 25) = 4.1, p < 0.05, η_p_^2^ = 0.24]. A post-hoc comparison (Tukey HSD test, MS = 0.01; df = 25.00) revealed lower accuracy performance in LTM group (p < 0.05) than controls (see Figure 1a). Groups did not differ regarding RTs of average emotions recognition [F (2, 25) = 1.84, p = 0.17, η_p_^2^ = 0.12]. Conversely, significant differences among groups were observed for disgust recognition [F (2, 25) = 3.97, p < 0.05, η_p_^2^ = 0.24]. Post-hoc comparisons showed significantly slower RT for controls than STM group (p < 0.5). No other differences were observed (see Figure 1b).

Empathy: Group differences were found in Personal distress subscale [F (2, 25) = 7.88, p < 0.01, η_p_^2^ = 0.38]. A post-hoc comparison (Tuckey HSD, MS = 13.53; df = 25.00) showed that both LTM and STM groups scored lower than controls (p < 0.01, for both). No other difference was observed (see Figure 1c).

Theory of mind (ToM): No group differences were observed [F (2, 25) = 1.10, p = 0.34, η_p_^2^ = 0.08] (see Figure 1d).

### Interoception

**Figure 2:**
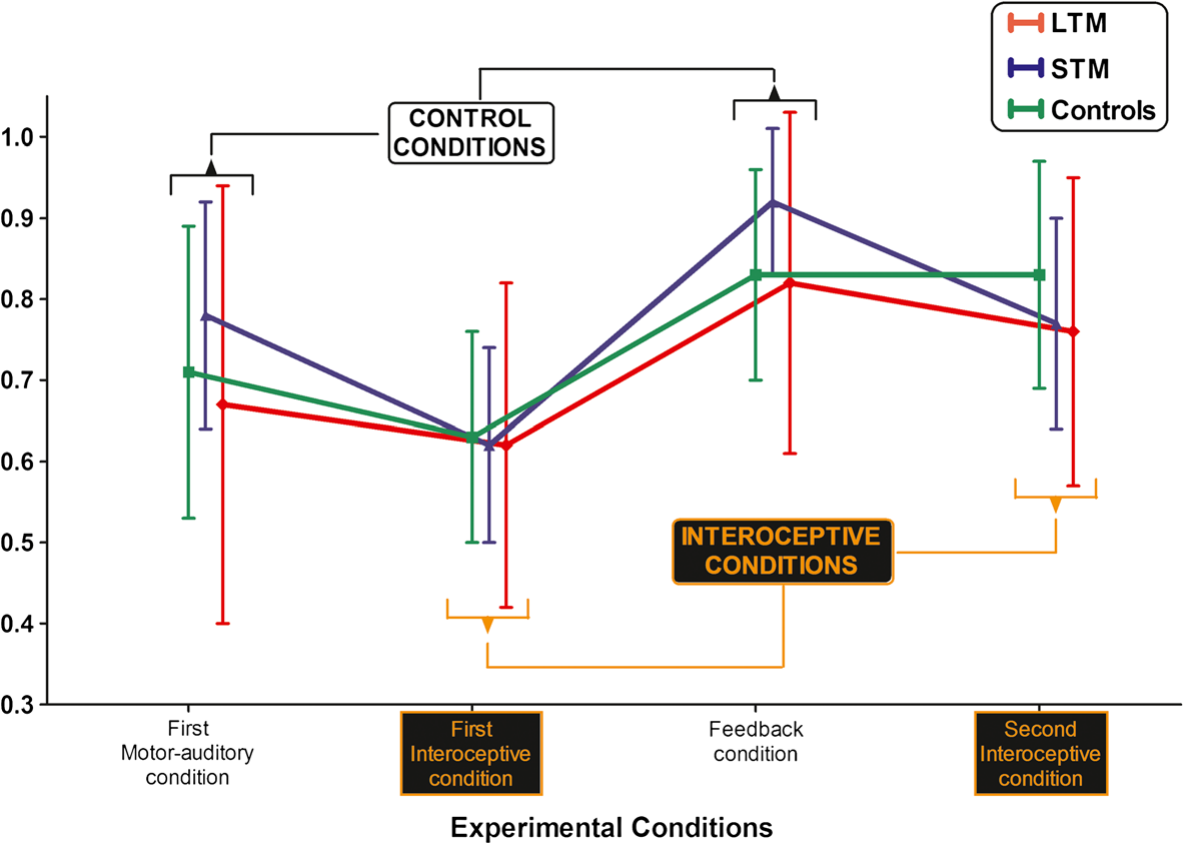
**Heartbeat detection task (HBD).** The Accuracy Index can vary between 0 and 1, with higher scores indicating better accuracy. No differences were found among groups in any condition. Vertical bars indicate standard deviation.

No group effects [F (2,25) = 0.57, p = 0.57, η_p_^2^ = 0.04] or condition × group interaction [F (6, 75) = 0.59, p = 0.72, η_p_^2^ = 0.04] were observed. Thus, there were no significant differences in the ability to track their heartbeats (interoceptive conditions) or an external cued heartbeat (motor and feedback conditions), in any of the four conditions (See Figure 2). Only an expected [12] and irrelevant effect of condition was observed (see Additional file 2: Table S2).

## Discussion

This is the first study assessing the influence of meditation practice both in cardiac interoception and in social cognition using a range of tasks. We selected a HBD task that avoids the possible interference of external stimuli [11] previously reported [5,9].

No differences in EF or demographic variables were observed. Related to mood and affective scales, controls showed higher STM (depression) and LTM (anxiety-trait) scores. These results might reflect the possible influence of skills acquired during meditation practice (without considering its length), such as stress coping and emotional regulation abilities, which could help to deal with anxiety and depression situations. These skills might modulate mood perception as more euthymic and positive [20].

Regarding interoception, we replicated negative results previously reported [5,9]. Body awareness includes one internal (viscera and blood composition) and one external stream (taste, smell, pressure sensations and pain [21]). Consequently, cardiac sensations might be considered as a basic modality of visceral perception that relies mostly on internal drive (the heart being an internal organ), which is why it would be more difficult to gain conscious inspection. Respiration is unique among interoceptive signals as it involves external pressure information from the nose and chest, and it is susceptible of voluntary control and straightforward conscious perception. During meditation, attention is commonly directed towards breathing [5], where more consistent results have been shown [2,7]. These findings suggest that cardiac perception might not be the most suitable index to reflect meditation influence on interoception.

Few group differences were observed in social cognition domains. The lower accuracy in disgust recognition found in LTM compared to controls might be related to their lower cardiac interoceptive sensitivity (given the common insular involvement for interoception and disgust recognition [22]). However, this is speculative because interoceptive differences were not significant among groups.

Both meditators’ groups showed significantly lower empathy scores compared to controls only in the personal distress subscale, an index of emotional contagion by others’ distress [23]. This is unsurprising since one of the aims of meditation is the regulation of responsiveness to stressors [24]. Finally, no difference in ToM was observed. Overall, despite the few differences reported, groups have similar social cognition performance suggesting that meditation practice in this study may not impact on these abilities.

Our study suffers from important limitations. First, the sample size should be increased to allow more informative analysis (i.e. correlations, multiple regressions) about the association among meditation, interoception and social cognition. However, it is worth highlighting that we reported preliminary data about interoception sensitivity measure with a novel method, and that previous research has employed similar sample size [9]. Second, further studies should cover a multidimensional interoceptive assessment (not only cardiac but also breathing, cardiac, visceral, etc.) and including both awareness and sensibility dimensions. Finally, groups’ homogeneity should be guaranteed by measuring variables that might bias visceral perception such as physical state, volume stroke, blood pressure and contractibility (Additional file 2: Table S2).

## Conclusion

In conclusion, no influence of meditation practice in cardiac interoception and related social cognition measures was observed. Based on the existence of diverse interoceptive signals, a more extensive assessment of each visceral source (other than cardiac one) may be necessary to disentangle the influence of meditation on interoceptive sensitivity.

## Abbreviations

HBD: Heartbeat detection task; LTP: Long term practitioners; STM: Short term meditators; MBSR: Mindfulness-based stress reduction; ToM: Theory of mind; EF: Executive functions; BDI: Beck’s depression inventory; STAI: State trait anxiety inventory; IFS: INECO frontal screening; IRI: Interpersonal reactivity index.

## Competing interests

All the authors declare that they have no competing interests with respect to this study or its publication.

## Authors’ contributions

MM and LS collected the data, statistically analyzed the data and wrote the first draft of the manuscript. BC was involved in the study conception and design, writing the protocol and contributed to the drafting of the manuscript. MR contributed in collecting the data and revising the final version of the manuscript. CG contributed to writing the final version of the manuscript. ACJ and MS contributed to revising the final version of the manuscript. FM contributed to revising the final version of the manuscript. AI is the head of our laboratory, was involved in the study conception and design and contributed to writing the final version of the manuscript. All authors read and approved the final manuscript.

## Authors’ information

Margherita Melloni, Lucas Sedeño as the first author.

## Supplementary Material

Additional file 1 **Methods.** In this Additional file 1 we provide a supplementary and detailed description of the materials and methods used in the study Click here for file

Additional file 2 **HBD additional results.** In this Additional file 2 we provide a supplementary description of others interoceptive results. Click here for file
