# Supplementary material for: Preliminary evidence about the effects of meditation on interoceptive sensitivity and social cognition
Source: Behav Brain Funct. 2013 Dec 23;9:47. doi: 10.1186/1744-9081-9-47 (PMC3878404; doi:10.1186/1744-9081-9-47)
Supplement: Additional file 1 — Methods. In this Additional file 1 we provide a supplementary and detailed description of the materials and methods used in the study [file 1744-9081-9-47-S1.docx]

**Methods**

**Subjects' selection criteria**

The short-term group included subjects that completed an eight week Mindfulness-Based Stress Reduction (MBSR) program developed by Dr. Jon Kabat-Zinn at the University of Massachusetts Medical Center [[1](#_ENREF_1)]. MBSR program has been assessed by three instructors of the Mindfulness Centre “Vision Clara” (<http://www.visionclara.org/> ): Clara Badino, a Yoga instructor trained by Dr. Jon Kabat-Zinn, Dr. Julio Laurindo, a psychiatrist trained by Chiara Badino and Martín Reynoso, a psychologist also trained by Chiara Badino. MBSR program follows the instruction that Kabat-Zinn’s (1990) MBSR program recommends and include teaching of meditation techniques in a weekly group setting of one hour duration and a home meditational and yoga practice guided by audio recordings of between 20 and 30 minutes every day. Subjects attended all weeks of the program. At the end of the training, participants completed a self-report questionnaire about their compliance with the home training. Table Suppl. 1 shows the similar adherence of all subjects to home practicing.

Participants from the long-term meditation group have previously participated in the MBRS program of the “Vision Clara” Mindfulness Centre and then continued their meditation practice as instructors or participants in other programs of the Centre. They fulfilled the following criteria: 1) be practitioners of mindfulness or vipassana meditation techniques; 2) reported at least one year of continued meditation practice; and 3) dedicated more than 60 minutes per week to meditation exercise (see Table Suppl. 2).

The control group consisted of individuals selected from a waiting list of the program who had never attended a formal yoga or meditation course.

**Neuropsychological and mood evaluation**

The Beck Depression Inventory-II (BDI-II) [[2](#_ENREF_2)] is a 21-item depression scale for assessing emotional, behavioral, and somatic symptoms. Items on the BDI-II are rated on four-point scales ranging from zero to three, with a maximum total score of 63. Higher scores indicate more severe depressive symptoms.

The STAI [[3](#_ENREF_3)] is a 40 item scale, which assesses both state and trait anxiety and represents well-validated and reliable self-report measures of dispositional and state anxiety. The scales for trait and state anxiety are made up of 20 items each; respondents are asked to indicate to what degree the items describe their dispositional and situational feelings on a four-point Likert-type scale (where 1 = “not at all” and 4 = “very much so”).

The Frontal Screening test (IFS) [[4](#_ENREF_4)], assesses 8 different domains of EF and has already been used to assess frontal performance in lesion patients [[5](#_ENREF_5)]. The IFS assesses frontal lobe function as an index of the following subtasks: Motor Programming, Conflicting Instructions, Verbal Inhibitory Control, Abstraction, Backwards Digit Span, Spatial Working Memory, and Go/No Go.

**Emotion Recognition**

Emotional morphing. This facial expression recognition task features six basic emotions (happiness, surprise, sadness, fear, anger and disgust) taken from the Pictures of Affect Series (Ekman and Friesen, 1976), which have been morphed for each prototype emotion and for a neutral state (Young et al., 1997). This procedure involved taking a variable percentage of the shape and texture differences between the two standard images 0% (neutral) and 100% (full emotion) in 5% steps (500 msec for each image). The 48 morphed facial stimuli were presented on a computer screen (in a random order) for as long as the subject took to respond by pressing the keyboard. Each participant was asked to respond as soon as they recognized the facial expression and then to identify it from a forced choice list of six options. In this task both accuracy of the emotion recognition and reaction times (RTs, calculated on both correct and incorrect participants’ responses) were measured.

**Interoceptive Measures**

Heartbeat detection task. We carried out a behavioral HBD that has already been described and validated in a previous work of our group. [[6](#_ENREF_6)]. In this task participants had to tap a computer key along with their heartbeat in different conditions. First, as a motor control condition, each patient was instructed to follow an audio recording of a sampled heartbeat. Next, they were told to follow their heartbeat with no external stimulation or feedback (intero-pre condition). They were then instructed to do the same while receiving simultaneous auditory feedback of their own heart provided through online EKG register (feedback condition). Finally, they were once again told to follow their own heartbeat without any feedback (intero-post condition). These four conditions provide us with a measure of merely audio-motoric performance (first condition), as well as with a cardiac interoceptive measure (second and fourth conditions), previous to and after the feedback condition, respectively. Thus, the subjects were able to implicitly compare their performance in the first interoceptive condition against this auditory cue (*feedback*) and consequently, it was expected that in the second interoceptive condition they adjust their performance according to that and perform it more accurately. ECG signal was recorded with an *ad-hoc* circuit composed of an amplifier AD620 and a band-pass filter (low 0.05 Hz, high 40 Hz) and then analogically fed to a laptop computer’s audio-card. Three Ag/Ag-Cl adhesive electrodes were placed to every participant in lead-II positions, together with headphones for audio stimuli delivery. The signal was processed on-line with a PsychToolbox [[7](#_ENREF_7)] script running on Matlab platform (MathWorks). External electrodes were used in the EEG setup (see below) to collect the ECG signal, which was processed in real time for peak detection and audio stimulation following the heartbeats. The output variables include:

a) **Total Correct Answers:** shows the total amount of each of the subject’s responses that correspond to his/her own heartbeat. Every motor response is compared within a specific time window around every recorded heartbeat; if the tap input is temporally locked within a time window for any heartbeat, that response is considered as correct (the time window is determined by the subjects’ heart rate: among 125 msec before and 750 msec after the beat, for a heart rate (HR) less than 69.76; among 100 msec before and 600 after, for HR between 69.75 and 94.25; and 75 msec before and 400 msec after, for HR higher than 94.25). The total sum of all the subject’s responses that fulfill this temporal criterion comes up to be this index.

b) **Recorded Heartbeats:** refers to the total amount of heartbeats recorded in each condition.

In order to compare subjects without the possible bias of heart rate’s differences among them, we calculated an Accuracy Score based on these two indexes. This score is a modified equation of the one proposed by Schandry for his heartbeat mental tracking method [8[8](#_ENREF_8)]. Schandry uses the total amount of mental heartbeats counted and the total number of heartbeats recorded. As we can discriminate from the total subjects’ responses the ones that are correct (according to the criterion explained in the first index), we used this more specific measure of the interoception sensitivity instead of the total sum of responses. Another difference with Schandry is that we calculated each index for every condition of the task. The accuracy equation we used is:

**1 - (Recorded heartbeats – ∑ Correct Answers)**

**______________________________________**

**Recorded heartbeats**

This interoceptive score can vary between 0 and 1, with higher scores indicating only small differences between correct answers and recorded heartbeats and thus better interoceptive performance.

**References**

1. Kabat-Zinn J, Nhat Hanh T: *Full Catastrophe Living: Using the Wisdom of Your Body and Mind to Face Stress, Pain, and Illness.* New York: Bantam Dell; 1990.

2. Beck A, Brown G, Steer R: *Manual for the Beck Depression Inventory-II.* San Antonio: The Psychological Corporation; 1996.

3. Spielberger CD, Gorsuch RL, Lushene RE: *Manual for the Stait-Trait Anxiety Inventory.* Palo Alto: Consulting Psychological Press; 1970.

4. Torralva T, Roca M, Gleichgerrcht E, Lopez P, Manes F: **INECO Frontal Screening (IFS): a brief, sensitive, and specific tool to assess executive functions in dementia.** *Journal of the International Neuropsychological Society : JINS* 2009, **15:**777-786.

5. Roca M, Parr A, Thompson R, Woolgar A, Torralva T, Antoun N, Manes F, Duncan J: **Executive function and fluid intelligence after frontal lobe lesions.** *Brain : a journal of neurology* 2010, **133:**234-247.

6. Couto B, Salles A, Sedeño L, Peradejordi M, Barttfeld P, Canales-Johnson A, Dos Santos YV, Huepe D, Bekinschtein T, Sigman M, et al: **The man who feels two hearts: Heartbeat detection, social cognition and emotional processing through different interoceptive pathways. In press.** *Social cognitive and affective neuroscience* 2013.

7. Brainard DH: **The Psychophysics Toolbox.** *Spatial vision* 1997, **10:**433-436.

8. Schandry R: **Heart beat perception and emotional experience.** *Psychophysiology* 1981, **18:**483-488.

**Table Suppl. 1: Short-term Meditators MSBR adherence.**

|  | **Average Home Practicing of the STM group** | |
| --- | --- | --- |
|  | **Training Days (during the week)** | **Training minutes per day** |
| **Week 1** | M= 6.25, SD= 0.70 (5-7) | M= 32.5, SD= 8.86 (20-40) |
| **Week 2** | M= 6.37, SD= 0.74 (5-7) | M= 32.5, SD= 8.86 (20-40) |
| **Week 3** | M= 6, SD= 0.75 (5-7) | M= 32.5, SD= 8.86 (20-40) |
| **Week 4** | M= 5.5, SD= 0.92 (4-7) | M= 30, SD= 10.69 (10-40) |
| **Week 5** | M= 5.75, SD= 1.03 (4-7) | M= 35, SD= 10.63 (10-40) |
| **Week 6** | M= 5.5, SD= 1.19 (4-7) | M= 35, SD= 10.69 (10-40) |
| **Week 7** | M= 5.5, SD= 1.06 (4-7) | M= 35, SD= 10.69 (10-40) |
| **Week 8** | M= 5.5, SD= 1.19 (3-7) | M= 32.75, SD= 10.60 (10-40) |

*M=mean;*

*SD=standard deviation*

**Table. Suppl 2: Long-term Meditators: description of meditation experience**

| Meditation techniques | Mindfulness (9 participants) // Vipassana ( 3 participants) |
| --- | --- |
| Years of practice | M = 4.35; SD = 2.17 (1.5−7.50) |
| Minutes of practice per week | M = 286.60; SD = 249.12 (80−961) |

*M=mean;*

*SD=standard deviation*
