# Supplementary material for: Preliminary evidence about the effects of meditation on interoceptive sensitivity and social cognition
Source: Behav Brain Funct. 2013 Dec 23;9:47. doi: 10.1186/1744-9081-9-47 (PMC3878404; doi:10.1186/1744-9081-9-47)
Supplement: Additional file 2 — HBD additional results. In this Additional file 2 we provide a supplementary description of others interoceptive results. [file 1744-9081-9-47-S2.docx]

**HBD additional results**

In HBD, significant differences were found in the Total Accuracy Index when comparing four conditions interactions within subjects [F(3,75)=11.85, p<0.01, η_p_^2^= 0.32]. Post-hoc comparisons (Tuckey HSD test, MS=0.2; df= 75) revealed higher scores of the feedback condition against the motor control condition (p<0.01) and also lower scores in the interoception pre-condition compared both to the feedback condition (p<0.01) and to the interoception post-condition (p<0.01).

It is expected that subjects’ performance varies between different conditions due to the changes in the task settings, as it has already been stated in a previous study of our group [[1](#_ENREF_1)]. In this way, participants adjusted their performance in the feedback condition compared to the first interoceptive one according to the auditory cues that were available. As a consequence of this feedback exposure, in the second interoceptive condition (when they had to follow again their heartbeats without any kind of external cue) they adjusted their performance, thus their accuracy is better than in the previous interoceptive condition. This pattern of performance improvement is what we interpreted as interoceptive learning.

**Heart-rate and heart-rate variability analysis**

*Introduction*

Given the possible influence of cardiodynamics variables on interoceptive sensitivity performance, we compared heart rate (HR) and heart rate variability (HRV) among groups. Our results showed that groups presented similar HR and HRV measures during interoceptive task, discarding the role of these variables as confounders.

*Data analysis*

The ECG signal recorded from each condition (with the exception of the motor one) was utilized to calculate heart rate and heart-rate variability. We imported beat-to-beat RR interval data (extracted from the ECG using Matlab platform) to the Kubios HRV [[2](#_ENREF_2)], an advance software for heart rate variability (HRV) analysis. This software automatically analyzed the HRV in both time and frequency domains. Given that the analysis was done for short times (2.5 min approx. for each condition), an autoregressive (AR) algorithm was used to calculate power spectral, accordance to previous recommendations [[3](#_ENREF_3)]. This algorithm generates a power spectral analysis with different frequency bands: high frequency (HF), low frequency (LF) and very low frequency (VLF). The HF component has been associated with the respiratory rhythm of heart period variability and is considered a marker of vagal modulation [[4](#_ENREF_4)]. The LF component reflects the rhythm corresponding to vasomotor waves present in heart period and arterial pressure variability and is a marker of sympathetic modulation [[4](#_ENREF_4)]. The LF/HF ratio has been suggested to mirror sympatho/vagal balance [[5](#_ENREF_5)]. We expressed these frequency components in normalized units (n.u.) that represent the relative value of each power component in proportion to the total power minus the VLF component [[4](#_ENREF_4), [6](#_ENREF_6)].

The Kubios HRV also gives a mean heart rate (HR) measure for each condition. Mixed repeated measured ANOVA was performed for HR and HRV, with a within-subject factor (the three last conditions of the HBD) and a between-subject factor (group).

*Results*

The statistical analysis of the HRV was performed on normalized units (n.u.) from the LF/HF ratio because this is a unique measure that allows comparing the similarity of sympatho/vagal balances from different samples. No group effects [F(2, 23)=2.08, p=0.15, η_p_^2^ =0.15], condition effect [F(2,46)=0.28, p=0.76, η_p_^2^ =0.01] or condition x group interaction [F(4,46)=0.12, p=0.97, η_p_^2^ =0.01] were observed.

Regarding heart rate, we found a tendency in group differences [F( 2,25)=3.14, p=0.06, η_p_^2^=0.20]: a post-hoc comparison (Tukey HSD test, MS=347 df=25.00) revealed a higher heart rate in long-term meditators (LTM) group (p=0.05) only when compared to short-term meditators (STM). This tendency disappeared in the condition x group interaction [F( 4,50)=2.12, p=0.09, η_p_^2^=0.14]. Results also showed no condition effect [F(2,50)=1.60, p=0.21, η_p_^2^=0.06].

Table Suppl. 3 shows the descriptive statistics of HRV and HR per condition.

*Discussion*

Both HR and HRV were measured due to their possible influence on interoceptive sensitivity performance.

HRV results showed no differences between groups and conditions, dismissing the consideration of these variables as a bias of our interoceptive results.

Concerning the HR, we found differences among groups that might influence interoceptive performance. However, regarding HR outcomes two issues should be considered. First, differences were found only when LTM and STM were compared, but not in the other possible comparisons. In consequence, HR might have not influenced the results of interoceptive sensitivity when LTM were compared with controls (C) or when STM were compared with C (these were the relationships we aimed to challenge). In addition, previous reports have shown that heartbeat perception seems to be more closely associated with volume stroke, blood pressure and contractibility rather than with the HR [[7-9](#_ENREF_7)].

*Limitations*

Several variables that might influence interoception sensitivity performance, as physical state and measures of the strength of the heartbeat (like volume stroke, blood pressure and contractibility) have been not assessed in our work. Although previous studies focusing on the relationship between interoception and mindfulness [[10](#_ENREF_10), [11](#_ENREF_11)] and also other paradigmatic research about visceral perception processing [[12-15](#_ENREF_12)] had not considered these variables yet, future research should include the assessment of these potential confounders in their evaluation protocol.

**Table. Suppl 3: descriptive statistics of HRV and HR per condition.**

|  | **Intero- pre condition** | | | **Feedback condition** | | | **Intero-post condition** | | |
| --- | --- | --- | --- | --- | --- | --- | --- | --- | --- |
|  | *Controls* | *STM* | *LTM* | *Controls* | *STM* | *LTM* | *Controls* | *STM* | *LTM* |
| **LF** | **56.57** (13.16)  *39.44-74.52* | **57.01** (11.65)  *40.45-75.72* | **65.49** (15.91)  *43.20-83.67* | **48.16** (23.77)  *14.70-87.05* | **56.10** (12.72)  *42.14-76.89* | **66.79** (15.28)  *36.18-89.52* | **53.13** (17.79)  *21.64-78.60* | **61.05** (13.87)  *43.69-80.53* | **61.43** (22.65)  *20.88-93.23* |
| **HF** | **43.42** (13.16)  *25.47-60.56* | **42.98** (11.65)  *24.27-59.54* | **34.50** (15.91)  *16.33 – 56.80* | **51.84** (23.78)  *12.95-85.30* | **43.90** (12.72)  *23.10-57.85* | **33.21** (15.26)  *10.48-63.82* | **46.87** (17.79)  *21.40-78.35* | **38.94** (13.87)  *19.46-56.31* | **38.57** (22.65)  *6.77-79.12* |
| **LF/HF** | **1.53** (0.86)  *0.65-2.92* | **1.51** (0.82)  *0.68-3.12* | **2.57** (1.72)  *0.76-5.12* | **1.57** (1.92)  *0.17-6.72* | **1.50** (0.93)  *0.73-3.32* | **2.78** (2.32)  *0.57-8.54* | **1.49** (1.13)  *0.27-3.67* | **1.93** (1.25)  *0.77-4.13* | **3.16** (4.15)  *0.26-13.76* |
| **HR** | **74.38** (9.35)  *60.16-88.23* | **67.38** (10.2)  *55.25-83.54* | **81.14** (11.87)  *66.79-103.02* | **74.74** (10.23)  *59.22-89.84* | **66.94** (9,96)  *54.58-84.28* | **78.13** (14.36)  *53.19-100.49* | **75.30** (9.83)  *59.08-89.09* | **67.39** (9.58)  *55.90-83.77* | **78.32** (13.56)  *58.51-100.56* |

**Subjects Average in Bold** // (SD between brackets) // *Min and max in italic.*

1. Couto B, Salles A, Sedeno L, Peradejordi M, Barttfeld P, Canales-Johnson A, Dos Santos YV, Huepe D, Bekinschtein T, Sigman M, et al: **The man who feels two hearts: The different pathways of interoception.** *Social cognitive and affective neuroscience* 2013.

2. Tarvainen MP, Niskanen JP, Lipponen JA, Ranta-Aho PO, Karjalainen PA: **Kubios HRV - Heart rate variability analysis software.** *Computer methods and programs in biomedicine* 2013.

3. Taskforce: **Heart rate variability: standards of measurement, physiological interpretation and clinical use. Task Force of the European Society of Cardiology and the North American Society of Pacing and Electrophysiology.** *Circulation* 1996, **93:**1043-1065.

4. Malliani A, Pagani M, Lombardi F, Cerutti S: **Cardiovascular neural regulation explored in the frequency domain.** *Circulation* 1991, **84:**482-492.

5. Eckberg DL: **Sympathovagal balance: a critical appraisal.** *Circulation* 1997, **96:**3224-3232.

6. Pagani M, Lombardi F, Guzzetti S, Rimoldi O, Furlan R, Pizzinelli P, Sandrone G, Malfatto G, Dell'Orto S, Piccaluga E, et al.: **Power spectral analysis of heart rate and arterial pressure variabilities as a marker of sympatho-vagal interaction in man and conscious dog.** *Circulation research* 1986, **59:**178-193.

7. Cameron OG: **Interoception: the inside story--a model for psychosomatic processes.** *Psychosomatic medicine* 2001, **63:**697-710.

8. Eichler S, Katkin ES: **The relationship between cardiovascular reactivity and heartbeat detection.** *Psychophysiology* 1994, **31:**229-234.

9. Schandry R, Bestler M, Montoya P: **On the relation between cardiodynamics and heartbeat perception.** *Psychophysiology* 1993, **30:**467-474.

10. Khalsa SS, Rudrauf D, Damasio AR, Davidson RJ, Lutz A, Tranel D: **Interoceptive awareness in experienced meditators.** *Psychophysiology* 2008, **45:**671-677.

11. Nielsen L, Kaszniak AW: **Awareness of subtle emotional feelings: a comparison of long-term meditators and nonmeditators.** *Emotion* 2006, **6:**392-405.

12. Critchley HD, Wiens S, Rotshtein P, Ohman A, Dolan RJ: **Neural systems supporting interoceptive awareness.** *Nature neuroscience* 2004, **7:**189-195.

13. Dunn BD, Galton HC, Morgan R, Evans D, Oliver C, Meyer M, Cusack R, Lawrence AD, Dalgleish T: **Listening to your heart. How interoception shapes emotion experience and intuitive decision making.** *Psychological science* 2010, **21:**1835-1844.

14. Pollatos O, Gramann K, Schandry R: **Neural systems connecting interoceptive awareness and feelings.** *Human brain mapping* 2007, **28:**9-18.

15. Schandry R: **Heart beat perception and emotional experience.** *Psychophysiology* 1981, **18:**483-488.
